# Sex-Biased Dispersal of a Frog (*Odorrana schmackeri*) Is Affected by Patch Isolation and Resource Limitation in a Fragmented Landscape

**DOI:** 10.1371/journal.pone.0047683

**Published:** 2012-10-18

**Authors:** Yu Wang, Amanda Lane, Ping Ding

**Affiliations:** 1 The Key Laboratory of Conservation Biology for Endangered Wildlife of the Ministry of Education, College of Life Sciences, Zhejiang University, Hangzhou, China; 2 Faculty of Veterinary Science, University of Sydney, Sydney, New South Wales, Australia; University of Kent, United Kingdom

## Abstract

Sex-biased dispersal is widespread in the animal kingdom and is affected by numerous factors including mating system, social factors and environmental conditions. Unlike birds and mammals, there is no common trend in amphibians and explaining the direction and degree of sex-biased dispersal in species-specific cases is difficult. We conducted a study on dispersal of the Chinese piebald odorous frog (*Odorrana schmackeri*) in a fragmented landscape associated with dam construction. Ten microsatellite loci were used to analyze 382 samples sourced from 14 fragmented ‘islands’. Assignment tests indicated a significant pattern of female-biased dispersal on one island with inconsistencies in the strength and direction of this pattern between nearby islands. The effects of four island attributes and two potential impact factors on the pattern of sex-biased dispersal were examined. We found that the extent of isolation from the mainland and the number of breeding sites both showed a negative correlation with female biased dispersal, such that the closer an island is to the mainland the more likely it is to display female biased dispersal, and the more breeding sites on an island the more male immigrants. Based on these results, we conclude that geographic isolation and limited breeding resources are the most likely explanation for the patterns of dispersal observed in this fragmented population of amphibians.

## Introduction

As a key function in life history, dispersal influences population dynamics, genetic structure and the persistence of populations [1]. Dispersal is influenced by intraspecific and interspecific interactions, the distribution and availability of resources, competitive ability and habitat variability [1], [2]. Asymmetric dispersal of the sexes, wherein one sex moves away from the natal habitat while the other remains, occurs as a result of differences in evolutionary pressures between genders [3]. Sex-biased dispersal is widespread among vertebrates and has been extensively studied in mammalian and avian lineages [2], [4]. Indeed it is with reference to data from birds and mammals that three alternative hypotheses were generated to explain differences in dispersal patterns between the sexes [5]. These hypotheses are: (1) avoidance of inbreeding, which suggests that the sex will disperse if it bears a greater cost from breeding with relatives [5]; (2) local mating competition, which predicts that the individuals of one sex will disperse to prevent competition with related individuals for mates [6]; and (3) local resource competition, whereby one sex disperses to minimize competition among relatives for a limiting breeding resources [6]. All three hypotheses predict male-biased dispersal and female philopatry in polygynous species (primarily mammals) [7]. While in monogamous species (primarily birds), only local resource competition is thought to drive female dispersal and the other two hypotheses cannot predict sex-biased dispersal [7].

These ecological theories for dispersal were formed with reference to mammals and birds, and while other taxa have been studied [8]–[10] it is to a considerably lesser degree. To date, research has not found any common trends in the pattern of sex-biased dispersal in amphibians [11]. In the previous studies of amphibians, two species exhibited female-biased dispersal [9], [12], four displayed male-biased dispersal [13]–[16] and three showed no significant differences between sexes [11], [17], [18]. Understanding the patterns of sex-biased dispersal in more amphibians is helpful to formulating conservation tactics under the current scenario of global decline [19]. Furthermore, in order to test the generality of ecological theories of sex-biased dispersal, formulated utilising data from mammals and birds, we must study a greater diversity of taxa including amphibians.

Although there are several potential hypotheses for the occurrence of sex-biased dispersal, which is the most appropriate explanation for a specific species is difficult to determine [20]. This is because the dispersal of an individual is not only affected by the mating system, but also by social factors and environmental conditions [20], [21]. The geographic scale can also be of importance, for example a social rodent (*Microtus arvalis*) showed a spatially-influenced pattern of sex-biased dispersal in which dispersal was male-biased at a fine scale but there was no pattern of gender imbalance at a larger scale [3]. Identifying the correct factors influencing a specific species will be helpful in understanding the evolutionary processes at work.

We investigate the pattern of sex-biased dispersal in the Chinese piebald odorous frog (*Odorrana schmackeri*) subjected to habitat fragmentation associated with dam construction. Land-bridge islands which formed as water rises or as a result of habitat loss [22], are a typical case of fragmentation and have the advantages of well-delineated boundaries and a homogeneous matrix [23] such that they can be considered to be natural ecological laboratories [24]. *Odorrana schmackeri* is widely distributed in People’s Republic of China. It inhabits the edges of forests and breeds in streams [25]. It is a sexually dimorphic species with females growing significantly larger than males. It is also a prolonged breeder with reproductive activity from May to October [25]. Male *O. schmackeri*, who have a lower reproductive investment, are able to mate with multiple females in each breeding season as females mature asynchronously [26]; whereas females tend to mate only once per season [27]. As *O. schmackeri* does not provide parental care, female adults, having the greater reproductive investment, must maximize reproductive success through mate choice [28].

In this study, we predict that sex-biased dispersal will be influenced by habitat fragmentation and/or other landscape factors. These results may give theoretical support to sex-specific conservation strategies for this species. Therefore, we aim to address the questions: (1) what is the pattern of sex-biased dispersal of *O. schmackeri* in a fragmented landscape, (2) have landscape features or other factors influenced the pattern of sex-biased dispersal in this scenario.

## Materials and Methods

### Ethics Statement

Our research on Chinese piebald odorous frog in Thousand Island Lake was approved by the Chinese Wildlife Management Authority and conducted under Law of the People’s Republic of China on the Protection of Wildlife (August 28, 2004).

**Table 1:** Geographic location of sample sites, sample sizes, island attributes and genetic diversity of *Odorrana schmackeri* populations per site.

	Coordination		Sample size								
Code	East	North	M	F	Area (ha)	Isolation (m)	PAR	SI	MAR	*H*_O_	*H*_E_
01	118°49′01.1′′	29°30′30.0′′	9	10	128.32	1121	0.013	4.279	6.50	0.53	0.68
02	118°56′02.8′′	29°31′37.0′′	11	30	47.98	996	0.016	3.064	8.80	0.50	0.68
03	118°55′51.6′′	29°32′06.0′′	12	16	32.29	980	0.021	3.025	8.30	0.53	0.72
04	118°54′09.4′′	29°32′03.1′′	4	6	30.68	569	0.024	3.718	5.20	0.47	0.64
05	118°50′22.2′′	29°29′23.4′′	20	17	26.78	556	0.046	6.657	7.90	0.45	0.70
06	118°53′46.7′′	29°32′09.1′′	11	10	22.93	435	0.017	2.248	8.00	0.53	0.70
07	118°54′25.7′′	29°34′11.9′′	27	19	15.64	925	0.033	3.698	9.00	0.43	0.69
08	118°55′40.6′′	29°31′21.1′′	7	12	4.93	543	0.040	2.517	8.10	0.63	0.77
09	118°53′32.8′′	29°33′54.1′′	17	11	4.46	982	0.034	1.995	8.00	0.42	0.73
10	118°52′33.9′′	29°33′25.4′′	15	8	4.29	856	0.041	2.371	8.80	0.57	0.80
11	118°49′40.6′′	29°31′16.5′	13	15	3.07	250	0.039	1.938	7.50	0.45	0.73
12	118°53′07.9′′	29°30′00.8′′	12	22	2.17	563	0.055	2.270	8.90	0.44	0.73
13	118°53′52.3′′	29°34′05.3′′	8	8	2.03	571	0.032	1.295	6.70	0.51	0.72
14	118°53′33.4′′	29°30′15.6′′	17	15	1.74	715	0.040	1.346	8.50	0.46	0.72

M, male; F, female; PAR, perimeter/area ratio; SI, shape index; MAR, mean allele richness;

*H*_O_, observed heterozygosity; *H*_E_, expected heterozygosity.

**Figure 1:**
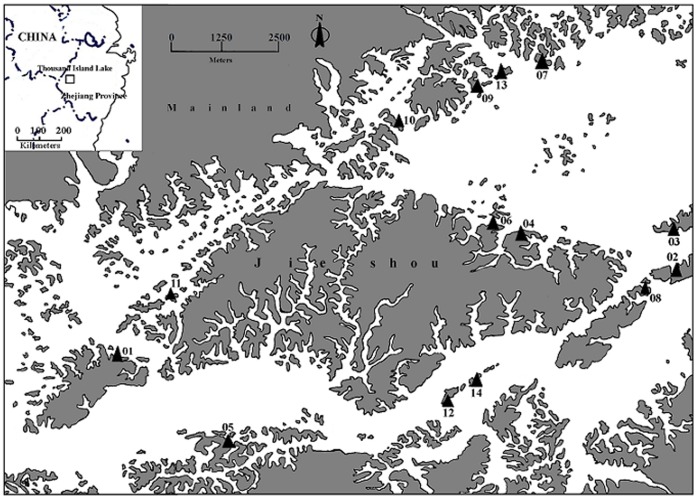
Map of study area and sampling sites. Black triangles show sampling distribution of *Odorrana schmackeri* in Thousand Island Lake of Zhejiang Province, China. Grey represents land, white indicates water.

### Study Area and Sampling

We conducted this study in Thousand Island Lake (29°22′–29°50′N, 118°34′–119°15′E), P. R. China. The lake was created in 1959 by damming and subsequently more than one thousand islands were formed. The vegetation on these islands consists of mixed subtropical deciduous and coniferous forest and the climate is typical of the subtropical monsoon zone. The temperature ranges from −7.6°C in winter to 41.8°C in summer. The mean annual precipitation is 1430 mm.

We collected frog samples via toe-clip during June and August (breeding season) of 2010 (Table 1, Figure 1). Quadrat method (100 m×3 m) was used to direct the sampling of frogs at the hydro-fluctuation zone of each island, which varies seasonally as a result of precipitation rates. Only tissue samples of adult frogs were utilized as the gender of subadult frogs which a snout-vent length less than four centimetres [25] could not be determined in *O. schmackeri*. Male adults were determined by the presence of nuptial pads during the breeding season. All samples were captured by hand, and tissue samples were stored in 95% ethanol for laboratory analysis.

### DNA Extraction and Microsatellite Genotyping

Genomic DNA was extracted from tissues using an adjusted proteinase K and phenol-chloroform ethanol precipitation protocol [29]. Twelve polymorphic microsatellite markers were used in polymerase chain reaction (PCR), including: Osch01, Osch02, Osch03, Osch04, Osch05, Osch06, Osch07, Osch08, developed by Wang & Ding [30] and Osch09, Osch10, Osch11, Osch12 (Table 2), developed in this study. The PCR conditions followed Wang & Ding [30]. Products from each PCR were run on an ABI 3730 automated sequencer (Applied Biosystems, Inc.) and genotyped with GeneMapper 3.7 software (Applied Biosystems, Inc.). To check the reliability of our results, 10% of amplified fragments were run at least twice in order to evaluate the repeatability of scoring, which was found to be consistent. Data were checked for scoring errors and null alleles using the program Micro-Checker version 2.2.3 [31]. The results showed the existence of null alleles at one locus Osch08, and indicated that locus Osch04 had >10% missing data. Consequently, we discarded these two primers and our analysis includes only the ten remaining reliable loci.

**Table 2:** Characterization of four polymorphic species-specific microsatellite loci isolated from *O. schmackeri*.

Locus	Repeat motif	Primer sequence (5′–3′)	Size range (bp)	*T*a (°C)	*N*_A_	*H*_O_	*H*_E_	GenBank nos.
Osch09	(GA)_10_	L: GAGTAGGAGGGTGTCAATG	106–122	55	6	0.357	0.569	JQ301792
		R: TGGTCCTCAAGCGTTAT						
Osch10	(TG)_10_(TC)_4_	L: TCACCAGAACAGCCAGAC	202–216	60	5	0.614	0.643	JQ301793
		R: TTGCTCAGGCATTCCAT						
Osch11	(TC)_10_A(TC)_3_	L: AAACCTCCGACCTCATCC	244–258	60	5	0.341	0.529	JQ301794
		R: CGTCTCCACCCTCATTACTT						
Osch12	(TG)_7_	L: TTCAGGCTCCACAACTT	165–189	58	11	0.412	0.781	JQ301795
		R: CTCCTCGTCTTTATTCACA						

*N*_A_, number of alleles; *H*_O_ and *H*_E_, observed and expected heterozygosity; *T*a, optimal annealing temperature.

### Island Attributes and Potential Impact Factors

For each sampling island, the island isolation (I) was measured by ArcGIS 10.0 (ESRI). This is the shortest distance from the sample site to the edge of mainland. Here we treated both the actual mainland and Jieshou Island as mainland, because Jieshou Island is >1,000 ha and the complement of plants and animals are similar to the mainland [32]. We also calculated island area (A) and perimeter (P) in ArcGIS 10.0. These two variables were used to calculate the perimeter/area ratio (PAR) and shape index, SI = P/(2×(π×A)^0.5^). PAR illustrates the relative amount of core habitat remaining on an island [33] and SI indicates island shape complexity [33]. The value of SI increases as the shape becomes more irregular and complex [23].

At the same time, two potential impact factors were analyzed: 1) sex ratio (SR) and 2) the number of breeding sites (BS). Sex ratio, the ratio of males to females in a population, was used to assess mating competition [34] under the assumption that it will be beneficial for frogs to disperse if an uneven sex ratio means that there are few opposite sex individuals on the natal island. The number of breeding sites per transect per island was used to evaluate the effects of resource competition on the observed sex-biased dispersal pattern. Though resource competition comprises both competition for survival (food) and reproduction (breeding site), we only used the number of breeding sites in our analysis as there are no other known sex-specific resources for this species (such as different water requirements or preferred habitat type). One prey type (Pine Moth larvae: *Dendrolimus punctatus* Walker) differs in consumption rates between the sexes (Wang *et al.* unpublished) but due to the small relative contribution of this food type, we do not consider food preference in our analysis. We calculated the sex ratio on each island by census of the number of males compared to females. During this sampling, the number of breeding sites was also counted in each 100 m×3 m transect. Breeding sites were identified and counted according to the known preferences of *O. schmackeri* which commonly breed in open sand hollows, or in rocky crevices at the hydro-fluctuation zone. These breeding sites were also checked for frogs and/or egg masses during census and sampling.

### Data Analysis

A Fisher’s exact test for Hardy-Weinberg Equilibrium (HWE) and genotypic linkage disequilibrium (LD) between all pairs of loci were estimated in GENEPOP version 4.0 [35]. The false discovery rate (FDR) method was applied to determine the significance of multiple comparisons [36]. Mean allele richness (MAR), observed and expected heterozygosity (*H*_O_, *H*_E_ respectively) for each population were calculated using CERVUS version 2.0 [37]. To evaluate the genetic difference within and among populations, we analysed molecular variance (AMOVA) by using software Arlequin version 3.5 [38].

Assignment tests were used to investigate the pattern of sex-biased dispersal in *O. schmackeri* in a fragmented landscape. This method was developed by Favre *et al.* [7] and followed the methods of Mossman & Waser [39] in GENALEX 6.4 [40]. This individual-based method can test populations or sites separately, rather than relying on the permutation of several populations. In brief, a log likelihood assignment test value is calculated for each individual, then the Assignment Index correction (*AIc*) score is calculated by subtracting the mean log likelihood of the population from an individual’s log likelihood score. For each population *AIc* values will average zero, while individuals with negative *AIc* scores are considered to have a higher probability of being immigrants. All 14 islands were assessed independently and the significance of sex-biased dispersal was examined using Mann Whitney U tests according to the difference between sexes in the frequency distribution of *AIc* scores. The significant level (0.05) was corrected by false discovery rate (FDR) [36] in order to control the multiple tests error.

To assess the influences of island attributes and potential impact factors on the pattern of sex-biased dispersal, we first quantified sex-biased dispersal at each island. The disparity *AIc* (d*AIc*) score was calculated by subtracting mean female *AIc* from mean male *AIc* [41]. A negative score indicates male-biased dispersal and a positive value is indicative of female-biased dispersal. Then we created a multivariate linear model: d*AIc*  =  a + bA + cI + dPAR + eSI + fSR + gBS, where a, b, c, d, e, f and g were the fitted parameters. To test the multicollinearity of all variables, we calculated the variance inflation factors (VIF). Only variables for which VIF values did not exceed a maximum threshold of 10 [42] were considered and analyzed. A backward stepwise analysis and information theoretic approach based on Akaike’s information criterion with second-order bias correction (*AICc*) [43] were used to choose the best fitted variables and evaluate the model. In order to estimate the contribution of each remaining parameter to disparity *AIc*, we used the variance partition method in the analysis [44]. All statistical analyses were using software R 2.15.0 [45]. This software was also used to calculate Chi-square (χ^2^) comparisons of sex ratios on each island.

## Results

A total of 451 samples were collected from 17 islands. However, only 382 tissue samples on 14 islands were utilized due to the presence of subadults on three islands. The mean allele richness ranged from 5.20 to 9.00, with average 7.87 (Table 1). The expected heterozygosity ranged from 0.64 in population 04 to 0.80 in population 10 (Table 1). After FDR correction, 67 out of 140 individual loci within populations showed significant deviation from Hardy-Weinberg Equilibrium and six pairs of loci in five populations were significantly out of linkage equilibrium. In the AMOVA test, the results showed that most of the molecular variance occurred within populations, while among populations there was explained only 4.77% (Table 3).

**Table 3:** The results of analysis of Molecular Variance (AMOVA).

Source of variation	Sum of squares	Percentage variation
Among populations	172.34	4.77***
Within populations	2680.37	95.23***

*** <0.001.

**Figure 2:**
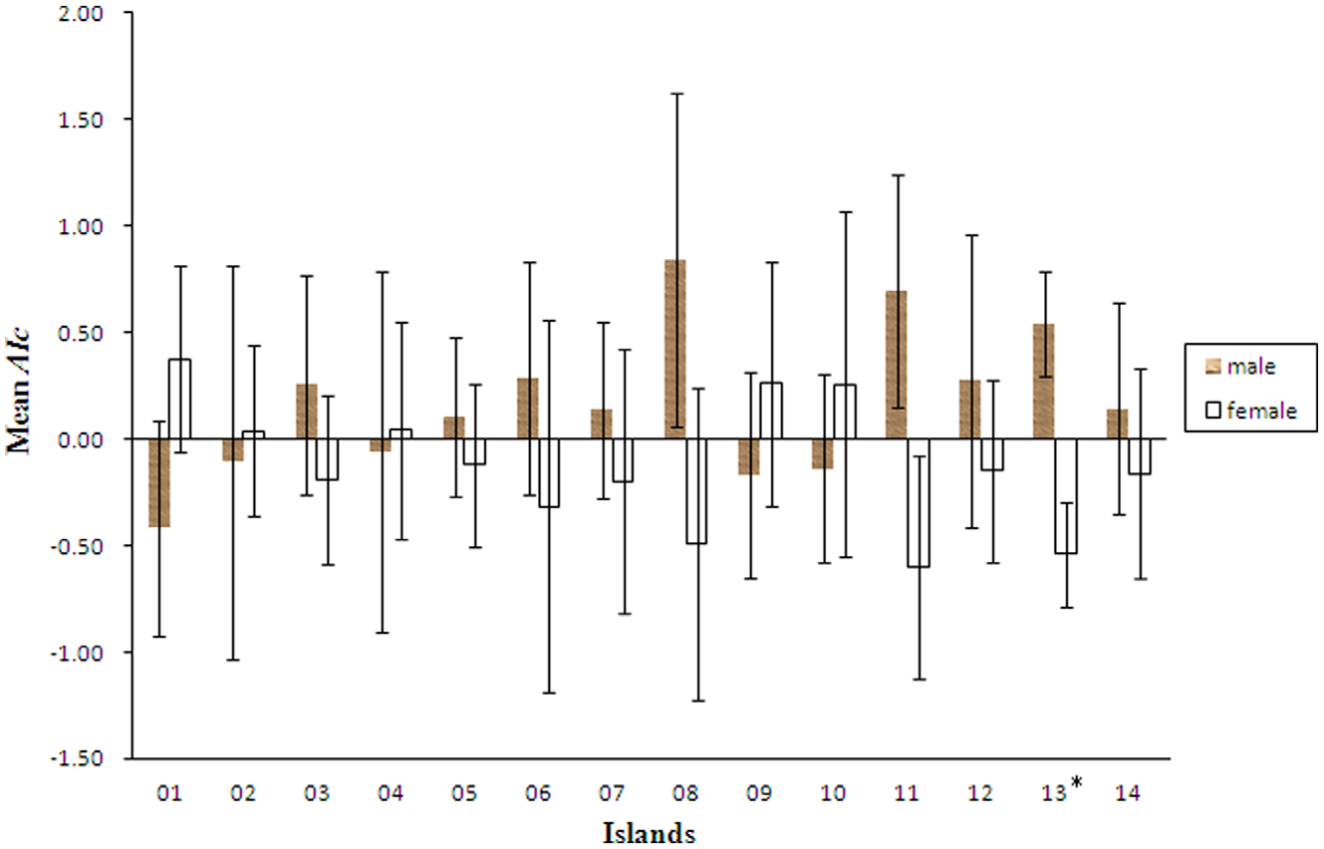
Mean *AIc* value for male and female of *Odorrana schmackeri.* The results demonstrate significant female-biased dispersal.^ *^ represent significant differences between sexes with Mann Whitney U test after false discovery rate correction.

Corrected assignment indices (*AIc*) indicate that nine of the 14 islands display female-biased dispersal (negative score for females, positive for males) while the remaining five islands display male-biased dispersal (negative score for males, positive score for females). Island 13 (P = 0.015) showed a significant degree of female-biased dispersal after FDR correction, whereas sex-biased dispersal on the other islands was not significant according to Mann Whitney U tests (Figure 2). The overall dispersal result when considering pooled data from all 14 island populations was male-biased, though this was not significant, as *AIc* values were similar at −0.016 and 0.014 for males and females respectively (P = 0.749).

**Figure 3:**
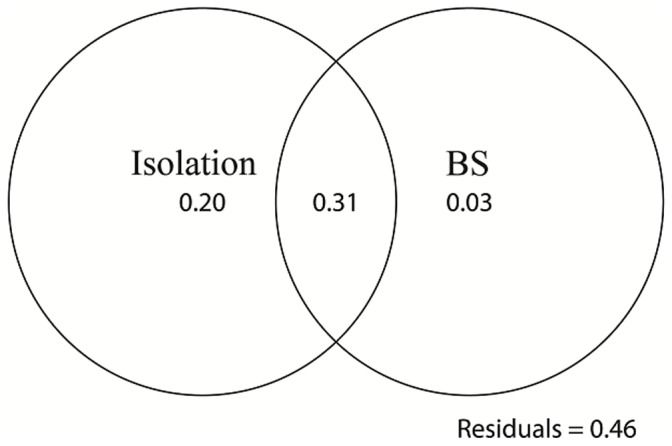
Variation partitions of effects of island isolation and the number of breeding sites on the disparity *AIc*. Island isolation explained more than the number of breeding sites and isolation play a more significant role in sex-biased dispersal. BS: the number of breeding sites.

All six parameters (A, I, PAR, SI, SR and BS) did not show evidence of multicollinearity. After backward stepwise analysis (Table S1), island isolation (I) and the number of breeding sites (BS) showed significant negative relationships with the disparity *AIc*. The average value of island isolation and the number of breeding sites are 718.71±257.76 (from 250 to 1121) and 62.86±39.39 (from 25 to 167), respectively. The best fit model was: d*AIc* = 1.630−0.001 * I−0.005 * BS (R^2^ = 0.543, *F* = 8.736, *P* = 0.005). The variance partitioning results showed that island isolation and the number of breeding sites explained 20% and 3% independently, but explained 31% together (Figure 3). The other four parameters displayed no correlation with disparity *AIc*. The average sex ratio value is 0.995±0.419 (ranged from 0.367 to 1.875) and Chi-square tests showed that there were no significant deviations from 1, except on island 2 (χ^2^ = 8.805, *P* = 0.003).

## Discussion

Our study demonstrates significant female-biased dispersal of *O. schmackeri* on an island within Thousand Island Lake, though there is inconsistency in this pattern with nearby islands showing non-significant female-biased and male-biased dispersal. The different patterns of sex-biased dispersal on each fragmented island imply that there may be different evolutionary pressures operating between the sexes at different locations and also illustrates that sex-biased dispersal is likely to be influenced by multiple factors and may not be shaped by mating system alone [3], [41].

The results of our stepwise linear model show that island area (A), perimeter/area ratio (PAR) and shape index (SI) are not associated with dispersal (disparity *AIc*). Island area is correlated with species richness but not dispersal in other systems [46], and whereas PAR and SI generally both indicate the quality of the remaining fragmented habitat [23], [33] they do not affect movement between islands in this case. However, island isolation (I) shows a significant influence on the disparity of *AIc* scores between sexes. Successful dispersal is not only determined by the movement ability of species, but also by the distance and matrix between patches. *Odorrana schmackeri* can successfully disperse to other islands, of a certain distance, by swimming in Thousand Island Lake. Hence the water is a semi-permeable barrier in this case, though it is considered a complete barrier for dispersal in other species [47]. It is of note that the success rate and potential distance travelled is expected to differ between sexes as the movement ability of females (the larger sex) is greater than that of males [25]. The negative relationship between disparity *AIc* and island isolation indicates that at sites closer to the mainland, females are more likely to be immigrants. This may be because more females than males are able to swim this distance. In contrast, sites far from the mainland tend to exhibit male-biased dispersal, though this is non-significant at *P*<0.05. In further remote islands, females may choose philopatry to breed with the most high quality local males as this involves less cost than benefits in attempting to disperse long distances. Males may immigrate from other populations to increase the probability of multiple matings with females as more females are located here. A similar relationship was concluded by Lane & Shine [41], wherein two amphibious sea snake species showed a negative relationship between *AIc* disparities and distance along a transect. One of these species (*Laticauda saintgironsi*) revealed intraspecific variation with significant female-biased dispersal in the island nearest to the mainland but significant male-biased dispersal in the farthest island, though the reasons for this pattern were not elucidated.

Another important factor which influences the pattern of sex-biased dispersal in *O. schmackeri* is the number of breeding sites. The negative correlation between disparity *AIc* and the number of breeding sites indicates that islands with a high number of breeding sites tend to have more male immigrants, and conversely islands with fewer breeding sites tend to exhibit female-biased dispersal. On islands with a high number of breeding sites, females can choose mates according to which males occupy a favorable breeding resource. In this case, males would likely immigrate from other populations as they can occupy an available breeding site and increase the chance of mating with multiple females, as this species is a prolonged breeder with asynchronous maturation [14]. On islands with fewer breeding sites, resident males who occupy the small number of breeding resources are expected to gain mates. Male immigrants may find it difficult to get mates as breeding sites are limited and they are not conferred the advantage of familiarity with these resources.

Sex ratio predicts the dispersing sex in theory [48], however we found no strong evidence for such a pattern in this system. According to the evolutionary stable strategy (ESS), the sex ratio commonly trends to one [49] but if there is mating competition, the equality of the sex ratio may break down [49]. Our results show that the sex ratio deviated significantly from one only on island 2 (χ^2^ = 8.805, *P* = 0.003), on which there were more females than males. In accordance with theoretical expectations, dispersal here favours male immigrants, however this pattern is not significant. The only island with significant sex-biased dispersal did not show a deviation of the sex ratio from one (χ^2^ = 0.000, *P* = 1.000 for island 13). Furthermore, sex ratio is not correlated with sex-biased dispersal overall, making mating competition an unlikely candidate to explain the observed pattern.

In the context of habitat fragmentation, comprehending the pattern and impact of a variety of landscape and other factors on sex-biased dispersal is important for understanding the evolutionary mechanisms at work and informing conservation efforts. Our results reveal that female-biased dispersal is negatively correlated with isolation from source locations and the number of breeding sites. It is important to note that these conclusions are based on a particular amphibian species and may not generalize across taxa.

## Supporting Information

Table S1 **The AICc values from backward stepwise analysis.** The result showed the lowest value when analysed these two factors isolation and the number of breeding sites.
(DOC)
