# Supplementary material for: Sex-Biased Dispersal of a Frog (Odorrana schmackeri) Is Affected by Patch Isolation and Resource Limitation in a Fragmented Landscape
Source: PLoS One. 2012 Oct 18;7(10):e47683. doi: 10.1371/journal.pone.0047683 (PMC3475718; doi:10.1371/journal.pone.0047683)
Supplement: Table S1 — The AICc values from backward stepwise analysis. The result showed the lowest value when analysed these two factors isolation and the number of breeding sites. (DOC) [file pone.0047683.s001.doc]

Table S1 The AICc values from backward stepwise analysis

| steps | models | AICc |
| --- | --- | --- |
| 1 | A+ I + BS + SI + PAR + SR | -16.02 |
| 2 | A + I + BS + SI + SR | -17.90 |
|  | I + BS + SI + PAR + SR | -17.78 |
|  | A + I + SI + PAR + SR | -17.67 |
|  | A + I + BS + PAR + SR | -17.53 |
|  | A + I + BS + SI + PAR | -17.02 |
|  | A + BS + SI + PAR + SR | -12.63 |
| 3 | I + BS + SI + SR | -19.76 |
|  | A + I + SI + SR | -19.52 |
|  | A + I + BS + SR | -19.15 |
|  | A + BS + SI + SR | -14.63 |
|  | A + I + BS + SI | -14.57 |
| 4 | I + BS + SI | -20.71 |
|  | I + SI + SR | -19.59 |
|  | I + BS + SR | -19.49 |
|  | SI + BS + SR | -15.62 |
| 5 | I + BS | -20.74^*^ |
|  | I + SI | -20.28 |
|  | SI + BS | -16.60 |
| 6 | I | -20.66 |
|  | BS | -16.44 |

A: area; I: isolation; PAR: perimeter/area ratio; SI: shape index; SR: sex ratio;

BS: breeding sites. * The best fitted model.
